# Thyroid transcription factor FOXE1 interacts with ETS factor ELK1 to co-regulate TERT

**DOI:** 10.18632/oncotarget.13288

**Published:** 2016-11-11

**Authors:** Martyn Bullock, Grace Lim, Cheng Li, In Ho Choi, Shivansh Kochhar, Chris Liddle, Lei Zhang, Roderick J. Clifton-Bligh

**Affiliations:** ^1^ Cancer Genetics Laboratory, Kolling Institute of Medical Research, Royal North Shore Hospital, Sydney, Australia; ^2^ University of Sydney, Sydney, Australia; ^3^ Storr Liver Centre, Westmead Millennium Institute for Medical Research, Westmead Hospital, Sydney, Australia; ^4^ Institute of Molecular and Experimental Medicine, School of Medicine, Cardiff University, Cardiff, UK; ^5^ Department of Endocrinology, Royal North Shore Hospital, Sydney, Australia

**Keywords:** thyroid cancer, BRAF-ERK pathway, FOXE1, ELK1, TERT

## Abstract

**Background:**

Although FOXE1 was initially recognized for its role in thyroid organogenesis, more recently a strong association has been identified between the *FOXE1* locus and thyroid cancer. The role of FOXE1 in adult thyroid, and in particular regarding cancer risk, has not been well established. We hypothesised that discovering key *FOXE1* transcriptional partners would in turn identify regulatory pathways relevant to its role in oncogenesis.

**Results:**

In a transcription factor-binding array, ELK1 was identified to bind FOXE1. We confirmed this physical association in heterologously transfected cells by IP and mammalian two-hybrid assays. In thyroid tissue, endogenous FOXE1 was shown to bind ELK1, and using ChIP assays these factors bound thyroid-relevant gene promoters *TPO* and *TERT* in close proximity to each other. Using a combination of electromobility shift assays, *TERT* promoter assays and siRNA-silencing, we found that FOXE1 positively regulated *TERT* expression in a manner dependent upon its association with ELK1. Treating heterologously transfected thyroid cells with MEK inhibitor U0126 inhibited FOXE1-ELK1 interaction, and reduced *TERT* and TPO promoter activity.

**Methodology:**

We investigated FOXE1 interactions within *in vitro* thyroid cell models and human thyroid tissue using a combination of immunoprecipitation (IP), chromatin IP (ChIP) and gene reporter assays.

**Conclusions:**

FOXE1 interacts with ELK1 on thyroid relevant gene promoters, establishing a new regulatory pathway for its role in adult thyroid function. Co-regulation of *TERT* suggests a mechanism by which allelic variants in/near *FOXE1* are associated with thyroid cancer risk.

## INTRODUCTION

Thyroid cancer is the most commonly occurring endocrine malignancy, accounting for 1% of all cancer diagnoses each year. The most common histological subtype is papillary thyroid cancer (PTC), a carcinoma of follicular cell origin, which accounts for 80% of thyroid malignancies. PTC demonstrates a strong genetic component, since it shows the highest relative risk (FRR = 8.60–10.30) in first degree relatives of probands among cancers not displaying Mendelian inheritance [1, 2].

FOXE1 (Forkhead Box E1), a Forkhead (FOX) transcription factor is essential for thyroid gland development [3–9], and is also required in the hormonal regulation of thyroglobulin (*TG*) and thyroid peroxidase (*TPO*) gene expression by the adult thyrocyte [10–12]. Recent genetic studies have identified germline allelic variation in and near *FOXE1* to be strongly associated with non-medullary thyroid cancer risk including single nucleotide variants rs965513[A] (56 kb upstream of *FOXE1*) [13–18] and rs1867277[A] (within its promoter) [19–21], and variation within the *FOXE1* polyalanine tract [22–24]; resolution of causal variants responsible for the association with thyroid cancer has been difficult due to strong linkage disequilibrium between all three variants. Nevertheless, these allelic variants were associated with altered FOXE1 expression in PTC tissues [25], whereas complete loss of FOXE1 expression is often found in anaplastic thyroid cancer (ATC) [26, 27]. Conversely, the ‘benign’ rs965513[G] allele has been associated with hypothyroidism [28] and altered free T3/free T4 balance [13]. Together, these converging lines of evidence strongly suggest that FOXE1 is important for maintaining normal thyroid differentiation even in the adult gland. However, as of yet, no mechanistic data exists to explain the association between FOXE1 and thyroid cancer risk.

**Figure 1:**
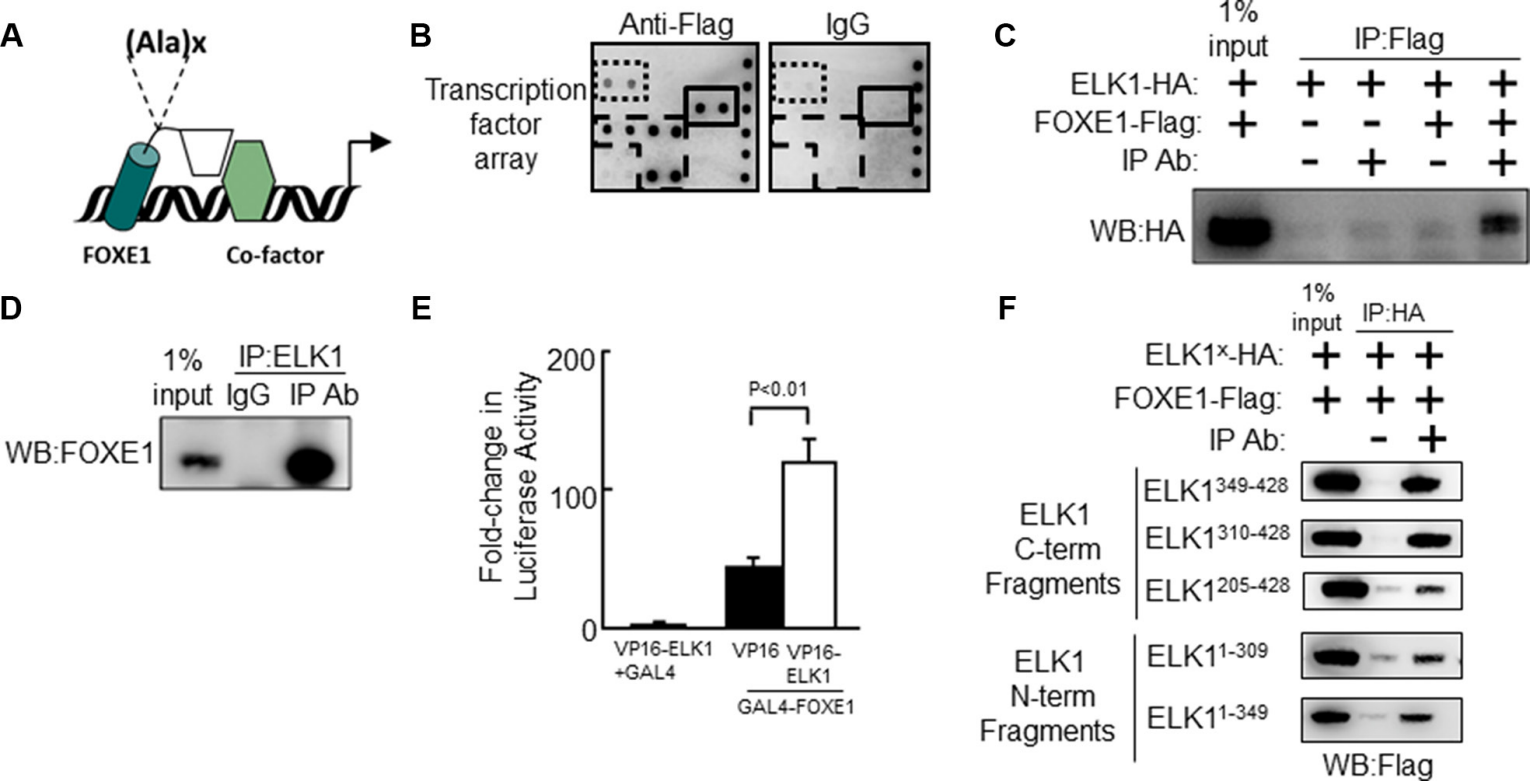
The Forkhead factor FOXE1 physically interacts with the ETS-factor ELK1 (**A**) Schematic of FOXE1 binding to target gene and interacting with a transcriptional co-factor. FOXE1 DBD is shown as a cylinder; its C-terminal domain is shown as a rhomboid; and a putative interacting co-factor is shown as a hexagon. The position of the FOXE1 polyalanine tract is shown, where x = 11–19 alanines. (**B**) Potential FOXE1-interacting partners detected with the TransSignal^™^ (Panomics) TF-TF interaction array-I. Nuclear extracts from NThy cells overexpressing FOXE1-Flag protein, were mixed with the TransSignal Probe mix, and immunoprecipitated using either an anti-Flag antibody or IgG isotype control. Duplicate spots corresponding to the ELK1 and c-REL are boxed with a solid line and dotted lines respectively. The other visible spots are signals for FOXF2, FOXD1 and FOXI1 binding sites, and are likely false-positives produced by FOXE1 directly binding the capture probe (boxed with a dashed line). (**C**) Validation of the FOXE1-ELK1 interaction by Co-IP of exogenous epitope-tagged proteins. NThy cells were transiently transfected with varying combinations of empty, FOXE1-Flag and ELK1-HA expression plasmids; immunoprecipitation was performed using an anti-Flag antibody (or IgG isotype control), and the western blot was probed with an anti-HA antibody. (**D**) Validation of the FOXE1-ELK1 interaction by Co-IP of FOXE1 and ELK1 proteins, endogenously expressed in thyroid tissue. Tissue lysate was immunoprecipitated with an anti-ELK1 (C-terminal domain) monoclonal antibody, and the western blot probed with an anti-FOXE1 monoclonal antibody. (**E**) Mammalian two-hybrid assay in HEK293 cells using transfected Gal4-FOXE1 and ELK1-VP16 and pGL5-luc reporter. Proteins were harvested 48 hrs post-transfection and reporter assays performed. Values are the the mean (± SD) of three experiments, each performed in triplicate, expressed as fold increase in luciferase activity relative to cells transfected only with reporter. (**F**) Mapping the location of the FOXE1-ELK1 interaction domain, by Co-IP of lysates from NThy cells expressing full-length FOXE1-Flag protein with various truncated mutant forms of ELK1-HA.

Recent studies have demonstrated that FOX proteins often regulate key pioneer functions via interaction with key transcription factors [29], dysregulation of which can cause cancer [30]. We reasoned that FOXE1 role in thyroid cancer might be explained by discovering its interacting partners and cognate transcriptional pathways (Figure 1A). We tested this hypothesis by searching for FOXE1 interaction partners from a panel of transcription factors, and found that the strongest signal was for the ETS (E26 transformation-specific) factor ELK1. Since ETS factors are already strongly implicated in thyroid carcinogenesis as the principal end-effectors of the BRAF (v-Raf murine sarcoma viral oncogene homolog B)-ERK (Extracellular Signal Regulated Kinase) signalling cascade [31, 32], we proceeded to validate FOXE1-ELK1 physical and functional association by several experimental approaches. Finally, since ETS factors have been shown to regulate *TERT* (Telomerase Reverse Transcriptase) in cancer [32–35], we specifically examined FOXE1-ELK1 co-regulation of this gene promoter.

## RESULTS

### FOXE1 physically interacts with ETS factor ELK1

Firstly, we sought to identify candidate FOXE1-interacting transcriptional cofactors using a medium throughput protein-DNA array technology. NThy-ori-3.1 (NThy) thyroid cells were transiently transfected with either empty vector (negative control) or plasmid expressing Flag-tagged FOXE1 protein (FOXE1-Flag). Forty-eight hours post-transfection the cells were harvested and nuclear fractions were prepared for subsequent analysis with Affymetrix TF-TF interaction arrays I and II (screening a total of 150 different transcription factors). Figure 1B shows the anti-Flag antibody versus IgG isotype negative control results for a sub-region of array I. Significantly elevated signals, reflective of FOXE1-binding, were observed for several transcription factors such as ELK1, c-REL, FOXF2, FOXD1 and FOXI1 (the FOX proteins being likely false positives due to FOXE1 directly binding to their capture probes). The ERK-regulated ETS factor ELK1 (Figure 1B) was a candidate of particular interest, given the importance of BRAF-ERK signalling as a driver of thyroid tumorigenesis.

The physical association of FOXE1 and ELK1 was validated using co-immunoprecipitation (Co-IP) experiments, using proteins initially from transfected cells and then from thyroid tissue. Whole cells lysates were harvested from NThy cells transiently co-expressing FOXE1-Flag and HA-tagged ELK1 (ELK1-HA), immunoprecipitated with an anti-Flag antibody, and then subjected to analysis by western blotting. Figure 1C is a representative western blot showing that ELK1-HA could only be precipitated with anti-Flag antibody when FOXE1-Flag was co-expressed. Similarly, western blots of reciprocal Co-IP experiments (immunoprecipitating with an anti-HA antibody) could only detect FOXE1-Flag when both proteins were co-expressed (dns).

To confirm that the observed ELK1/FOXE1 interaction was not an artefact in these over-expression models, we next performed Co-IPs from thyroid tissue for endogenously expressed proteins. Figure 1D shows a representative western blot demonstrating that ELK1 was co-immunoprecipitated using an anti-FOXE1 antibody, but not with the corresponding IgG isotype control. The reciprocal experiment (immunoprecipitating with an anti-ELK1 antibody) yielded a similar result (dns).

We also confirmed physical interaction between FOXE1 and ELK1 in mammalian two-hydrid assays. Figure 1E shows that a construct containing the Gal4 DNA-binding domain (DBD) joined with the C-terminus of FOXE1 (amino acids 164–373) weakly activated the Gal4-reporter gene (pGL5-luc) alone, but co-transfection with a second construct containing full-length ELK1 tagged with the VP16 activation domain resulted in strongly enhanced transactivation.

Having established that the FOXE1 C-terminus is capable of binding ELK1, we next sought to identify the relevant interaction domain(s) of ELK1. Further Co-IP experiments were performed with a series of truncated ELK1-HA proteins in which previously characterised ELK1 domains were deleted; including the ETS DBD, SRF (serum response factor) interacting (S), transcriptional repressor (R), MAPK (mitogen activated protein kinase) docking (M) and transcriptional activation (A) domains (Supplementary Figure S1) [36, 37]. Altogether, the following truncations were generated: (1) amino-acids 1–309 (containing DBD, S and R domains), (2) 1–349 (DBD, S, R and M), (3) 205–428 (S, R, M and A), (4) 310–428 (M and A) and (5) 349–428 (A). Unexpectedly, we found that all ELK1 truncations tested could be co-precipitated with FOXE1-Flag (Figure 1F). This would suggest the existence of multiple interaction motifs through which FOXE1 may bind with ELK1. However, ELK1 is also known to homodimerize in solution via its own DBD [38]. Thus, it is also possible the DBD-containing truncations could be precipitated indirectly by forming dimers with endogenous full-length ELK1. Of note, C-terminal fragments encompassing amino acids 310–428 consistently demonstrated a more robust interaction with FOXE1 as compared with regions spanning amino acids 1–310 (Figure 1F). Interestingly, these truncations lack the transcriptional repressor domain (R domain), which by a sumoylation-dependent mechanism can disrupt ELK1’s interaction with cofactors [37].

### ELK1 recruits FOXE1 to the TERT promoter

A significant proportion of thyroid cancers harbour oncogenic mutations with the *TERT* promoter. These mutations generate *de novo* ETS-factor binding sites that mediate *TERT* transactivation in response to oncogenic BRAF-ERK-signalling. We therefore considered whether FOXE1 is recruited to the *TERT* promoter via interacting with ELK1.

**Figure 2:**
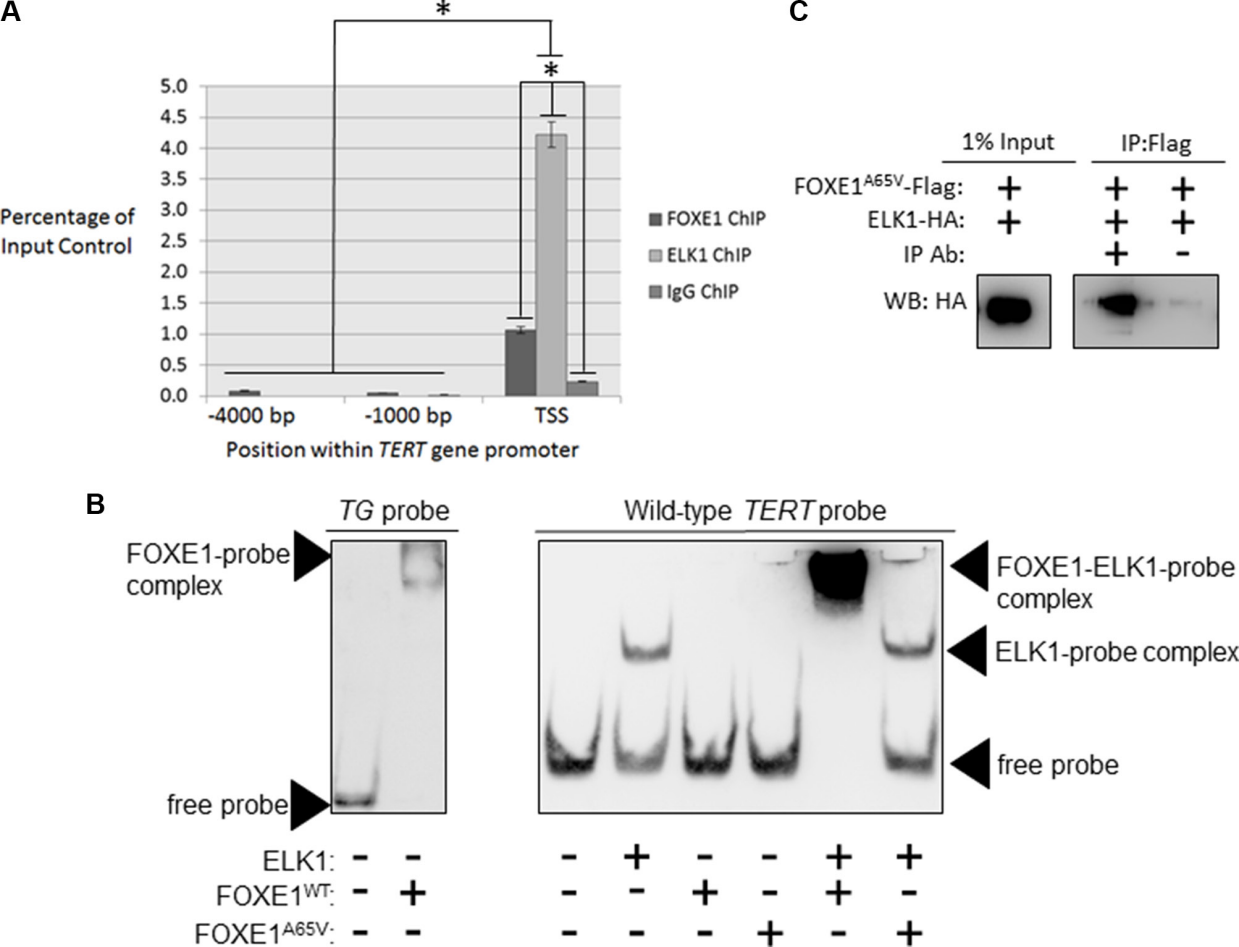
FOXE1 and ELK1 interact with the *TERT* gene promoter (**A**) Detection of FOXE1 and ELK1 binding at the *TERT* gene promoter by ChIP. Sheared formaldehyde-fixed chromatin was isolated from thyroid tissue and then immunoprecipitated with monoclonal antibodies raised against human FOXE1 and ELK1 proteins. ChIP DNA was amplified by real-time qPCR using primers specific for the proximal *TERT* promoter and two negative control regions located 1000- and 4000-bp upstream of the TSS. Enrichment of transcription factor binding was calculated as a percentage of the input DNA control. Values are the mean average and SD of three independent experiments. Significant enrichments over IgG controls are highlighted (*p < 0.01, Student’s *t-test*). (**B**) Measurement of the DNA-binding affinity of FOXE1 and ELK1 for the *TERT* promoter by EMSA. Varying combinations of purified FOXE1-Flag, FOXE1^A65V^-Flag and ELK-HA proteins were incubated with biotinylated *TERT* DNA-probe, resolved on a 6% polyacrylamide gels, and transblotted onto nylon membrane. (**C**) Determination of whether the FOXE1^A65V^ mutant and ELK1 can interact by Co-IP of exogenous epitope-tagged proteins. NThy cells were transiently transfected with varying combinations of empty, FOXE1^A65V^-Flag and ELK1-HA expression plasmids; immunoprecipitation was performed using an anti-Flag antibody (or IgG isotype control), and the western blot was probed with an anti-HA antibody.

*In silico* analysis (PROMO using version 8.3 of TRANSFAC [39, 40]) did not find a canonical FOX-binding site within the proximal *TERT* promoter (RYAAAYA [41]). Nevertheless, using formaldehyde-fixed chromatin isolated from thyroid tissue, chromatin immunoprecipitation (ChIP) revealed a 4.6-fold enrichment of FOXE1 (*p <* 0.01) in the same region of the *TERT* promoter (-151 to +11-bp relative to the TSS) that is specifically bound by ELK1 (Figure 2A). Supporting the specificity of this interaction, neither FOXE1 nor ELK1 DNA-binding could be detected in two upstream regions of the *TERT* promoter (located −1000 and −4000 bp relative to the TSS).

To determine whether FOXE1 directly binds to the *TERT* promoter, we conducted electromobility shift assays (EMSAs) using a biotinylated-probe corresponding to the promoter region identified in our ChIP experiments. We firstly confirmed that FOXE1 protein isolated from transfected HEK293 cells was able to bind its previously described cognate response element within the *TG* promoter [42] (Figure 2B, at left). We then incubated ELK1, FOXE1 or both with the *TERT* probe: this was readily bound by ELK1, but unexpectedly was only shifted by FOXE1 when ELK1 was also present (Figure 2B, middle). The same results were obtained when the *TERT* probe was mutated to contain the C228T or C250T variants that are found in thyroid cancer (Supplementary Figure S2). These results suggest that FOXE1 is only indirectly recruited to the *TERT* promoter via its interaction with ELK1, and that this indirect recruitment is not affected by cancer-associated mutations in the *TERT* promoter. Interestingly however, a DNA-binding mutation (p.Ala65Val) [3] in FOXE1 abrogated its interaction with *TERT*-bound ELK1 (Figure 2B), although it was still able to bind ELK1 in solution (Figure 2C). This suggested that FOXE1 may bind a non-consensus site within the *TERT* promoter but only after binding with ELK1.

**Figure 3:**
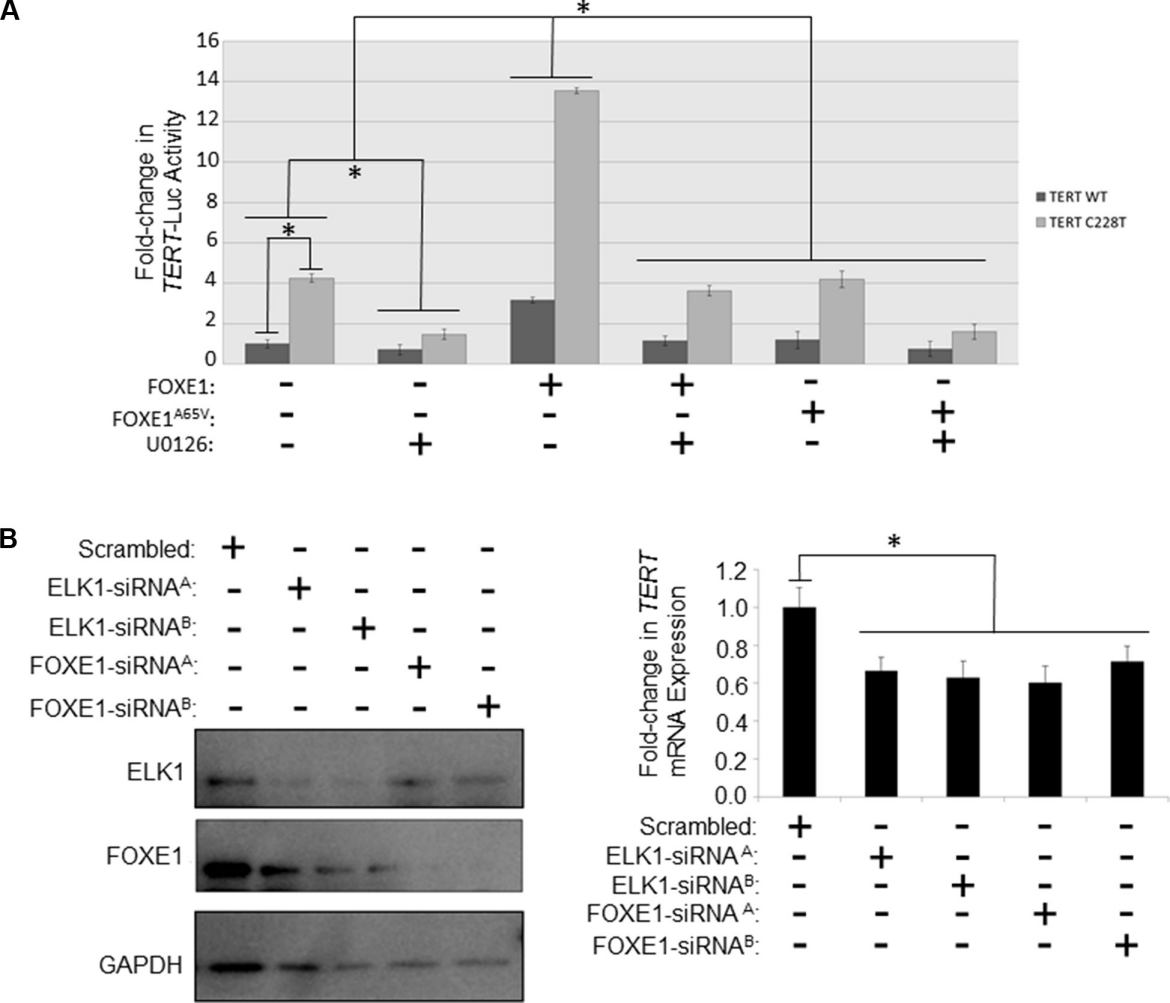
FOXE1 regulates *TERT* transcription in thyroid cancer cells (**A**) Determination of the transcriptional activity of FOXE1 upon the *TERT* gene promoter. KTC1 cells were transiently transfected with either wild-type or C228T *TERT*-luc, and different combinations of FOXE1-Flag, FOXE1^A65V^-Flag or empty Flag expression plasmids. Twenty-four hours post-transfection the cells were treated for a further 24 hours with 10 μM U0126 or vehicle, prior to whole cells lysates being harvested for luciferase reporter assays. Luciferase results are the mean (± SD) of three experiments, each performed in triplicate, expressed as fold change in luciferase activity relative to empty vector transfected cells. (**B**) Measurement of the changes in *TERT* mRNA transcription in response to depleting FOXE1 and ELK1 proteins. SW1736 cells were transiently transfected with FOXE1/ELK1 specific-siRNA (or scrambled siRNA control), then RNA and protein harvested from the cells 48 hrs later. FOXE1 and ELK1 levels were ascertained by western blotting, whilst *TERT* mRNA expression were quantified by real-time qRT-PCR. Significant changes are highlighted (*p < 0.05, Student’s *t-test*).

We then examined whether FOXE1 regulated *TERT* transcription in thyroid cells, and whether this effect was similar on native and mutant *TERT* promoters. For these experiments, we chose KTC1, which is a PTC cell-line; although similar results were also found in SW1736 (ATC) and NThy (non-tumorigenic thyroid) cell-lines (Supplementary Figure S3). As shown in Figure 3A, overexpression of FOXE1 stimulated both wild-type and C228T-mutated *TERT* promoters by 3.8 and 4.3-fold respectively compared with their respective empty vector-transfected controls. MEK inhibitor U0216 inhibited transactivation of the *TERT* reporter gene to a similar degree in either the presence or absence of FOXE1 (Figure 3A). Consistent with our findings in DNA-binding studies noted above, mutant FOXE1^A65V^ did not transactivate the *TERT* reporters (Figure 3A).

To confirm that *TERT* is regulated by endogenously expressed FOXE1, siRNA-mediated knockdown experiments were performed in SW1736 cells that express both FOXE1 and ELK1 (our own unpublished observations). *FOXE1*-targeting siRNA successfully depleted these cells of FOXE1 protein (Figure 3B, at left) and this was associated with a 28–40% reduction in *TERT* expression assessed by qRT-PCR (Figure 3B, at right). Similar repression of *TERT* was also detected when these cells were depleted of ELK1 protein (Figure 3B). However, simultaneous depletion of both factors caused significant cell death, to such an extent that the effect upon *TERT* expression could not be measured.

### MEK inhibition in thyroid cancer cells disrupts the FOXE1-ELK1 interaction

**Figure 4:**
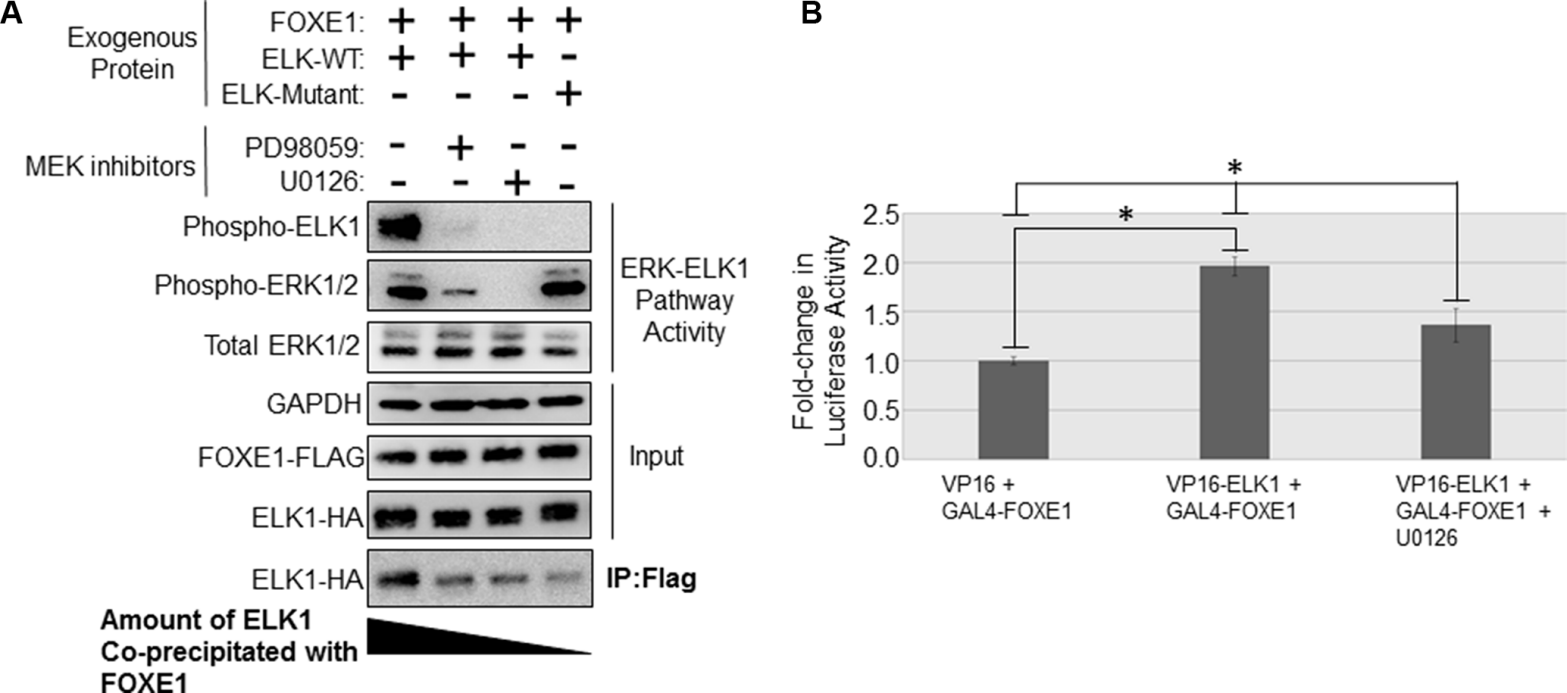
MAPK inhibition in thyroid cancer cells disrupts the binding of FOXE1 to ELK1 (**A**) Determination of the effect of MEK inhibition upon the FOXE1-ELK1 interaction. SW1736 cells were transiently transfected with various combinations of FOXE1-Flag and WT/mutant ELK-HA, treated with 10 μM MEK inhibitor or vehicle control, and then immunoprecipitation was performed using an anti-Flag antibody, and the western blot probed with an anti-HA antibody. (**B**) Mammalian two-hybrid assay in SW1736 cells using transfected Gal4-FOXE1 and ELK1-VP16 and pGL5-luc reporter. Twenty-four hours post-transfection, the cells were treated with 10 μM U0126 or vehicle control for a further 24 hrs, prior to the harvest of protein lysates and subsequent reporter assay. Values are the the mean (± SD) of three experiments, each performed in triplicate, expressed as fold increase in luciferase activity relative to cells transfected only with reporter. Significant changes are highlighted (*p < 0.05, Student’s *t-test*).

As noted above, treatment with a MEK inhibitor reduced FOXE1-mediated transactivation of the *TERT* reporter gene. To determine whether this was due to loss of physical interaction between FOXE1 and ELK1, we performed Co-IP assays using lysates from transfected SW1736 ATC cells containing the BRAF^V600E^ oncogene. As shown in Figure 4A, inhibition of the MEK/ERK activity partially inhibited (by approximately 20%) the interaction between FOXE1 and ELK1 in solution. This effect was seen following treatment with two different MEK inhibitors, and so was likely to be a specific effect of MEK inhibition. Conversely, when all known phospho-accepting Ser/Thr [49] (shown in Supplementary Figure S1) were mutated in ELK, this ERK-unresponsive mutant ELK1-HA also showed a significant reduction in its ability co-immunoprecipitate FOXE1-Flag (Figure 4A). Similar results were also obtained in KTC1 and NThy cells (dns). These data suggest that phosphorylation of ELK1 positively regulates its interaction with FOXE1. We explored this further in mammalian two-hybrid assays. As shown in Figure 4B, the interaction between Gal4-FOXE1 and VP16-ELK1 in regulating pGL5-Luc within SW1736 cells was diminished by 31% after treatment with U0126, consistent with the result from solution binding assays. Again, similar results were also obtained in KTC1 and NThy cells (dns).

### FOXE1-ELK1 interaction is also functionally relevant for TPO promoter activity

**Figure 5:**
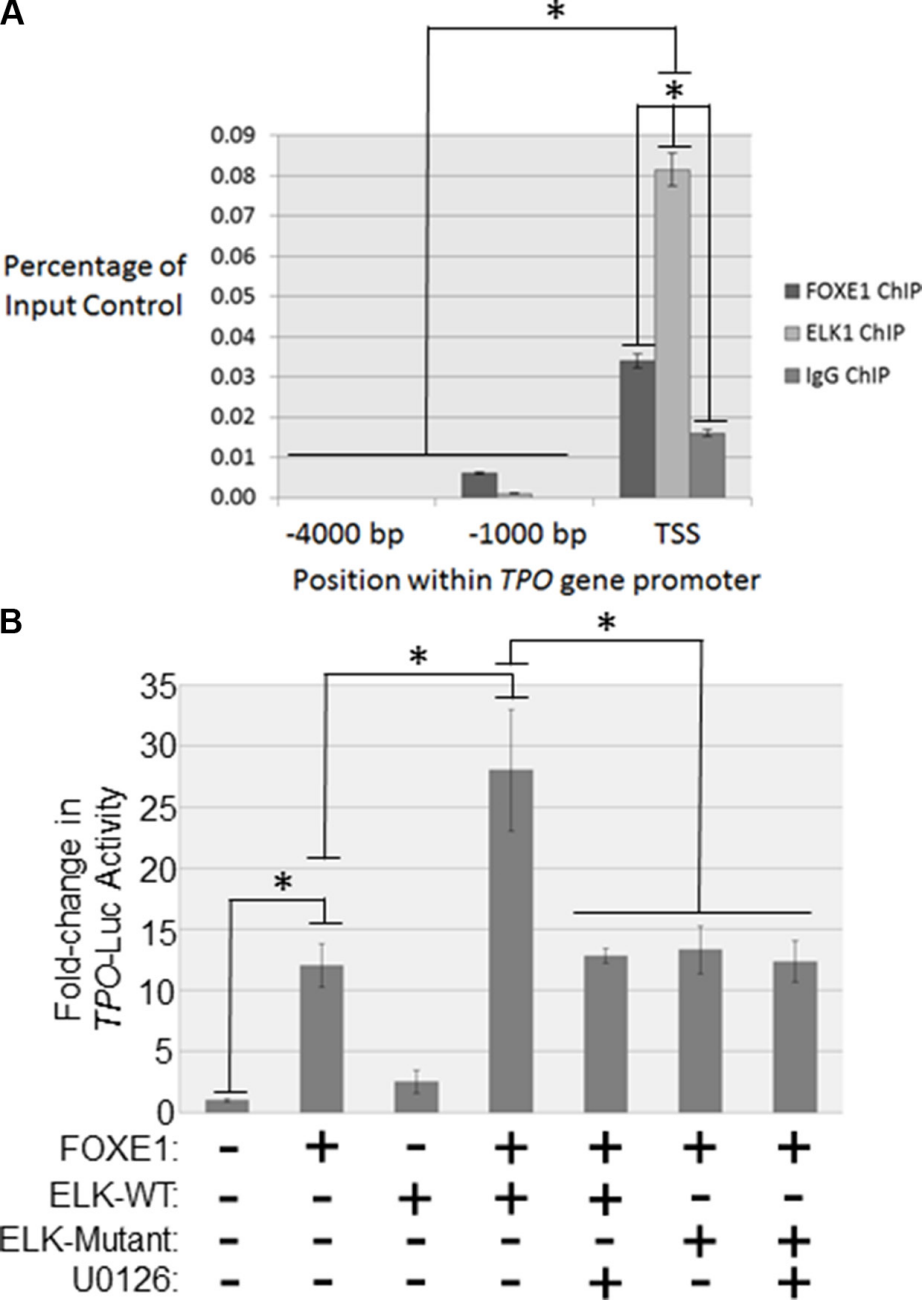
FOXE1 and ELK1 interact with the *TPO* gene promoter (**A**) Detection of FOXE1 and ELK1 binding at the *TPO* gene promoter by ChIP. Sheared formaldehyde-fixed chromatin was isolated from thyroid tissue and then immunoprecipitated with monoclonal antibodies raised against human FOXE1 and ELK1 proteins. ChIP DNA was amplified by real-time qPCR using primers specific for the proximal *TPO* promoter and two negative control regions located 1000- and 4000-bp upstream of the TSS. Enrichment of transcription factor binding was calculated as a percentage of the input DNA control. Values are the mean average and SD of three independent experiments. Significant enrichments over IgG controls are highlighted (*p < 0.01, Student’s *t-test*). (**B**) Characterizing FOXE1-ELK1 mediated regulation of the *TPO* gene promoter. NThy cells were transiently transfected with *TPO*-luc and different combinations of FOXE1, ELK1, mutant ELK1 or empty expression plasmids. Twenty-four hours post-transfection the cells were treated for a further 24 hrs with 10 μM U0126 or vehicle, prior to whole cells lysates being harvested for luciferase reporter assays. Luciferase results are the the mean (± SD) of three experiments, both performed in triplicate, expressed as fold increase in luciferase activity relative to empty vector transfected cells. Significant changes are highlighted (*p < 0.05, Student’s *t-test*).

Next we examined by ChIP whether FOXE1 and ELK1 were co-bound to the human *TPO* promoter, in a region orthologous to the well-characterized FOXE1-responsive element in the rat *TPO* promoter (−177 to −23 bp relative to the transcriptional start site) [10–12]. Our *in silico* analysis (PROMO using version 8.3 of TRANSFAC) also revealed this region to harbour predicted ETS factor binding sites. ChIP assays were performed using formaldehyde fixed chromatin isolated from thyroid tissue [26]. As expected we detected FOXE1 binding within close proximity to the *TPO* TSS (2.1-fold enrichment over the IgG negative control, *p <* 0.01) (Figure 5A). In contrast, two upstream promoter regions without predicted FOX-binding sites (−1000 and −4000 bp relative to the TSS) showed no enrichment for FOXE1-binding (Figure 5A). In agreement with our hypothesis that FOXE1 and ELK1 functionally interact on the *TPO* promoter, we also detected enrichment for DNA-binding of ELK1 (5.1-fold enrichment over IgG negative control, *p <* 0.01) close to the *TPO* TSS. Again, no significant enrichment of ELK1 binding was observed in the upstream promoter regions (Figure 5A).

We then investigated whether ELK1 affected FOXE1-stimulated transcription from the human *TPO* promoter. Overexpression of either FOXE1 or ELK1 in NThy cells stimulated *TPO* reporter activity by 12 and 2.5-fold respectively, relative to empty vector control (Figure 5B). In support of our hypothesis that these factors co-operate to enhance gene transcription, simultaneous co-expression of ELK1 and ELK1 enhanced TPO reporter activity by 2.3-fold, relative to FOXE1 alone. Consistent with our hypothesis that MEK-ERK inhibition disrupts FOXE1-ELK1 interaction, this enhancement of promoter activity was lost following treatment with a MEK inhibitor. Similarly, an ERK-unresponsive ELK1 mutant was also found not to be capable of enhancing FOXE1-stimulated reporter activity.

### Expansion of the FOXE1 Polyalanine Tract

**Figure 6:**
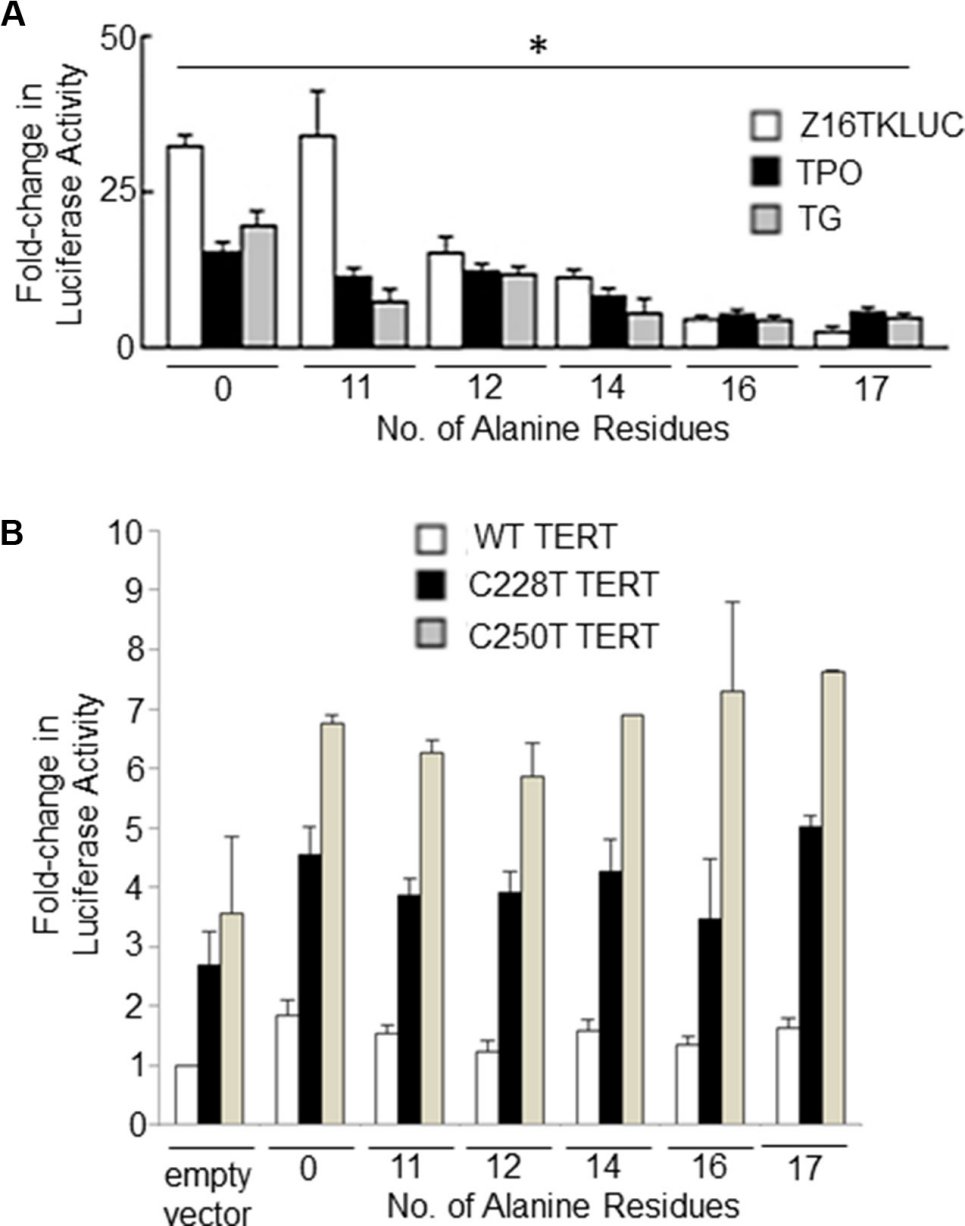
The impact of polyalanine tract length upon FOXE1 mediated transcriptional regulation NThy cells were co-transfected with FOXE1-Flag expressing plasmids of varying polyalanine tract size (0-17Ala) and (**A**) reporter plasmids containing known FOXE1-responsive elements – the native *TPO* and *TG* promoters and a synthetic Z16TKluc; or (**B**) reporter plasmids containing the wild-type and mutant (C228T or C250T) *TERT* gene promoters. Twenty-four hours post-transfection, cell lysates were harvested and luciferase assays performed (*p < 0.01 between polyalanine variants on each reporter gene, One-Way ANOVA).

In our previous study we demonstrated using gene reporter assays that the FOXE1^16Ala^ –thyroid encoded by the cancer risk allele was less transcriptionally active than the FOXE1^14Ala^ (encoded by the major allele in all populations) on thyroid-gene specific promoters [23]. Here, we extended these experiments to encompass the majority of polyalanine tract alleles observed in normal populations (11–17 alanine residues). Figure 6A shows the results of gene reporter assays which demonstrate an inverse correlation between polyalanine tract length and the ability of FOXE1 to transactivate three different FOXE1-responsive promoters (the native human *TPO* and *TG* promoters, and a synthetic construct Z16TKLUC [3]). In mammalian two hybrid experiments, we observed that increasing polyalanine tract length also had an inhibitory effect upon Gal4-FOXE1 and VP16-ELK1 stimulated promoter activity (Supplementary Figure S4). In contrast, the length of the polyalanine tract did not moderate FOXE1 transactivation of either wild-type or mutant (C228T or C250T) *TERT* gene promoters (Figure 6B).

## DISCUSSION

Little is known about the role of FOXE1 in the adult thyroid gland, or about those mechanism(s) by which variants in or near *FOXE1* (all of which are in tight linkage disequilibrium) are associated with thyroid cancer risk. We hypothesized that identifying FOXE1 transcriptional partners would shed light on the mechanism of its association with thyroid cancer. In this paper we have identified that FOXE1 binds with ELK1 and functionally co-regulates *TERT* in thyroid cells. Our data highlights a new biological pathway by which FOXE1 binds with ELK1 to alter transcriptional function of thyroid genes.

Other FOX family members have previously been noted to regulate gene transcription via binding with ETS factors, including FoxC2/Etv2 [43] and FoxO1/Ets1 [43, 44]. In the case of FoxC2/Etv2 co-regulation of vascular endothelial promoters, composite *cis-*acting motifs containing a consensus FOX DNA binding element upstream of a consensus ETS element have been identified in a large number of endothelial gene promoters [43]. In our study, the well-studied ETS response element in *TERT* was not obviously nearby a consensus FOX response element; nevertheless, we were able to show that FOXE1 bound this *TERT* enhancer when ELK1 was also present and that this binding was abolished by a DNA-binding mutation in FOXE1. Our data are most consistent with a mechanism by which ELK1 changes the *TERT* promoter structure in some manner that enables recognition of a non-consensus element by FOXE1; such a mechanism has also been reported for FOXM1 for which non-consensus binding sites were identified throughout the genome that nevertheless required an intact Forkhead DBD [45]. Diversity of Fox domain DNA binding has also been identified using evolutionary approaches, suggesting a model whereby conformational rearrangement of the DBD through specific co-partner interaction changes its sequence recognition motifs [46].

The particular role of ETS factors in malignancy has been known for some time: activation/overexpression of ELK1 has been implicated in the pathogenesis of several malignancies including breast and bladder [47, 48] and the TCF (ternary complex factor) subfamily, consisting of ELK1, ELK3 and ELK4, are particularly sensitive to ERK-mediated phosphorylation [49]. Our study is the first to identify a possible role for FOX:ETS interaction in malignancy.

The importance of finding a new mechanism of *TERT* regulation is underscored by the plethora of emerging data regarding this oncogene in malignancy. Somatic mutations in the *TERT* promoter were first identified in melanoma [50, 51] and have been observed at high frequency in multiple cancer types, including those of the thyroid, central nervous system, and bladder [52–54]. The two hot-spot mutations (chr5:1,295,228C > T, “C228T”, and chr5:1,295,250C > T, “C250T”) both create a putative consensus binding site (GGAA) for ETS transcription factors that in turn drive increased *TERT* expression [50]. We and others have previously shown that MEK inhibition successfully blocked transactivation of *TERT* promoter constructs containing these oncogenic mutations [55, 56]. In the present study we have now identified that FOXE1 co-regulates *TERT* via interaction with ELK1, and that MEK inhibition partially abrogates this interaction. Our data raise the opportunity for discovering new therapies directed at *TERT* inhibition via targeting this interaction.

Finally, with respect to the association between variants in/near *FOXE1* and mechanisms of thyroid cancer risk, our data provides a new mechanism by which FOXE1 can affect cancer development via *TERT* upregulation. It remains unclear how these FOXE1 variants affect function: one study proposed that rs1867277 functionally alters binding of USF1/USF2 transcription factors within the FOXE1 proximal promoter [19]; other work has proposed that rs965513 identifies a group of long-range enhancer elements that regulates FOXE1 expression [57]. Our own previous work suggested that a longer FOXE1 polyalanine tract (FOXE1^16Ala^) is transcriptionally impaired upon the *TPO* and *TG* gene promoters [23], and here we demonstrate that this negative relationship holds true for a wide-range of naturally occurring polyalanine tract variants. We also demonstrate that increasing length of the polyalanine tract length negatively impacts upon the ability of FOXE1 to interact with ELK1. This concept that impaired FOXE1 function drives thyroid oncogenesis is also supported by recent description of a germline missense *FOXE1* mutation in one family with non-medullary thyroid cancer [58], and also by finding somatic *FOXE1* missense mutations in sporadic thyroid cancer [59]. However, in this study we were unable to show an effect of the polyalanine tract expansion *per se* on FOXE1-mediated transcriptional regulation of *TERT*. Thus, the functional effects of the polyalanine tract appears to be promoter-context dependent, and it may influence oncogenic pathways via, as of yet, unidentified FOXE1 regulated genes. Further studies using manipulation of endogenous FOXE1 polyalanine tracts, and global gene expression analysis, will be required to explore this hypothesis in more detail.

Overall, our work sheds new light on the role of FOXE1 in thyroid cancer susceptibility, and the transcriptional partnership between FOXE1 and ELK1 opens new therapeutic possibilities, either via targeting of FOXE1-ELK1 binding (in a similar manner to the targeting of FOXM1 which is currently undergoing preclinical investigation [60]), or via modulating of ELK1 phosphorylation (e.g. as we have shown here using MEK inhibition).

## MATERIALS AND METHODS

### Plasmid constructs

Plasmids were generated using PCR-cloning and site-directed mutagenic techniques (PCR primer sequences are provided in Supplementary Table S1). For Co-IP experiments, both full-length and truncated ELK1 coding sequences were cloned into the NotI-XhoI site of pCMV-HA-N vector (Takara Bio Inc, Japan). The full-length FOXE1 coding sequence was cloned into EcoRI-BamHI site of p3XFlag-CMV-7.1 (Sigma-Aldrich, St Louis, MO, USA). For mammalian two-hybrid experiments, the pACT-ELK1 construct was generated by cloning the ELK1 coding sequence into the BamHI-KpnI site of the pACT vector (Promega, Madison, WI, USA). The pBIND-FOXE1 construct was created by cloning the coding sequence of the FOXE1 C-terminus into the BamHI-KpnI site of the pBIND vector (Promega). For gene reporter experiments, a 474 bp region of the human *TERT* promoter (−391 to +83 relative to the TSS) was cloned into the KpnI-HindIII sites of the pGL3-basic vector (Promega). Generation of the pGL3-TPO reporter has been described previously [61]. Preparations of purified plasmid DNA for transfections were prepared using Qiagen’s Qiafilter plasmid maxi kit, according to the manufacturer’s instructions (Qiagen, Hilden, Germany).

### Cell culture

HEK293 cells were grown in DMEM, and NThy-ori-3.1 (non-tumorigenic thyroid), SW1736 (*BRAF^V600E^*, *TERT^C228T^*, p53-null ATC) and KTC1 (*BRAF^V600E^*, *TERT^C250T^*, p53 positive PTC) cells were grown in RPMI. All growth media was supplemented with 10% fetal calf serum, 50 U/ml penicillin, 50 μg/ml streptomycin, and cells were maintained in 5% CO2 concentration at 37^°^C. The growth media of the KTC1 cell-line was additionally supplemented with 1% non-essential amino acids (all reagents from Thermo Fisher Scientific, Waltham, MA, USA). The identity of each cell-line was confirmed by STR profiling (CellBank Australia, Sydney, Australia). Furthermore, mutational status of the cells was confirmed by Sanger sequencing.

### MEK inhibition

Cells were serum-starved for 24 hrs prior to be treated with either 10 μM U0126 or 50 μM PD98059 (Merk KGaA, Darmstadst, Germany), or DMSO (Sigma-Aldrich) vehicle control. For Co-IP and luciferase reporter experiments, cells were treated for 1 hr and 24 hrs, respectively, prior to cell-lysis.

### siRNA knockdown

The day before transfection, SW1736 cells were plated in a 6-well culture plate at density of 2 × 10^5^ cells per well. Cells were transiently transfecting with Roche’s XtremeGene siRNA transfection reagent (Roche, Basel, Switzerland); with 160 pmoles of FOXE1/ELK1 specific siRNA or AllStars negative siRNA control (Qiagen). Forty-eight hours after transfection, total RNA was extracted using an RNeasy Plus mini kit (Qiagen).

### Western blot

Firstly, the protein concentration of each whole cell/nuclear lysates was determined using the RC DC Protein Assay kit (Bio-Rad, Hercules, CA, USA), and then 10 mg of each protein sample was resolved by SDS-PAGE and electroblotted onto Hybond ECL membrane (GE Healthcare Life Sciences, Chicago, IL, USA). The membrane was incubated overnight at 4^°^C with either anti-FLAG (M2) (Sigma-Aldrich, #F1804); anti-HA (Cell Signalling Technology, Danvers, MA, USA; #3724); anti-phospho ELK1 (Ser383; CST #9181); anti-phospho ERK (Thr202/Tyr204; CST #4370); anti-total ERK (CST, #4695); or anti-GAPDH (CST, #2118); using dilutions recommended by the manufacturer. The blot was then probed for 1 hr at room temperature with a 1:10,000 dilution of goat anti-rabbit/mouse, IgG HRP-linked antibody and developed using the ECL-Prime Western Blotting Detection Reagent (GE Healthcare Life Sciences). Densitometric analysis of western blots was performed using a multi-gauge imaging system (FujiFilm, Tokyo, Japan).

### RT-PCR

Contaminating genomic DNA was removed using DNAse I and RNeasy Plus mini kit (Qiagen), and then cDNA was generated using Superscript III reverse transcriptase (Thermo Fisher Scientific). Gene expression was determined using Taqman probes (Thermo Fisher Scientific) and run on an ABI7900HT. Ribosomal 18S expression was used as a normaliser in all experiments.

### Mammalian two-hybrid and luciferase reporter assays

The day before transfection, HEK293 and SW1736 cells were plated in a 24-well culture plate at density of 1 × 10^5^ and 5 × 10^4^ cells per well respectively. For luciferase reporter assays, cells were transiently transfected with Roche’s XtremeGene HP liposomal-based transfection reagent; with 500 ng firefly luciferase reporter plasmid, 50 ng renilla luciferase reporter plasmid and 100 ng of cDNA expression plasmid (or empty expression vector control). For mammalian two-hybrid assays, cells were transfected with 1 μg pGL5-luc reporter and 100 ng each of the Gal4-FOXE1 and VP16-ELK1 expression plasmids. Transfected cell were incubated for 24 hr, before they were lysed in 100 μl of 1X Promega passive lysis buffer (Promega).

### Transcription factor interaction array

The TranSignal™ TF-TF Interaction Arrays I and II (Affymetrix, Santa Clara, CA, USA) was used to screen for interactions between FOXE1 and 150 different transcription factors. Briefly, nuclear protein was extracted from NThy-ori-3.1 cells transiently transfected with a 3XFlag-FOXE1 expression plasmid. Nuclear lysate was incubated with the biotin-labeled, double-stranded oligonucleotide probes provided, allowing the transcription factor cis-elements to bind the Flag-tagged FOXE1 protein in the sample extract. An immunoprecipitation was then performed using a mouse monoclonal anti-Flag (M2) antibody (Sigma-Aldrich, F1804), to pull out the transcription factor cis-elements interacting with FOXE1. To control for non-specific binding an immunoprecipitation with IgG (Thermo Fisher Scientific, #31903) was performed in parallel. Non-specific binding was then washed away. The cis-elements were bound to FOXE1, and the anti-Flag (M2) antibody were then eluted and hybridized to the TranSignal Array membrane (one membrane per sample and a IgG negative control), and interactions detected using a horse-radish peroxidase (HRP)-based chemiluminescent detection system.

### Co-immunoprecipitation

Whole cell lysates were prepared with chilled protein lysis buffer (0.1% Triton-X100, 150 mM NaCl, 1 mM EGTA, 1 mM EDTA and 20 mM Tris-HCl, pH 7.5), containing 1X Halt protease and phosphatase inhibitor cocktail (Thermo Fisher Scientific). For overexpressed exogenous protein, 300 μl of lysate was prepared from approximately 1 × 10^6^ transfected NThy-ori-3.1 cells. Endogenous proteins were harvested in 1 ml of lysis buffer isolated from approximately 2 × 10^7^ cells. For each immunoprecipitation, the lysates were combined with 50 μl Dynabeads^®^ Sheep anti-Mouse or anti-Rabbit IgG (Thermo Fisher Scientific) combined with 1 μg of precipitating primary antibody (or IgG negative control), and incubated overnight at 4^°^C. Flag-tagged FOXE1 and HA-tagged ELK1 were precipitated with anti-Flag (M2) (Sigma-Aldrich, F1804) and anti-HA (Cell Signalling Technology, #3724) antibodies respectively. Endogenous FOXE1 and ELK1 were precipitated with anti-FOXE1 [EPR6843] (Abcam, Cambridge, UK; #ab134129) and anti-ELK1 [E277] (Abcam, #ab32106) respectively. The following day, the beads were subjected to six 1 ml washes with ice-cold lysis buffer. Finally, the purified proteins were eluted in 20 μl laemmeli buffer incubated at 95^°^C for 10 mins.

### Chromatin immunoprecipitation

Samples of frozen Graves’ thyroid tissue were obtained from the Neuroendocrine Tumor Bank located at the Kolling Institute, Royal North Shore Hospital. Approval for use of these samples was obtained from the local institutional human research ethics committee.

Formaldehyde cross-linked chromatin was prepared from Graves’ thyroid tissue using a protocol adapted from the methodology developed by the Myers laboratory [62]. Briefly, 50–100 mg ground frozen tissue was fixed in phosphate buffered saline (PBS) containing 1% formaldehyde, incubated at room-temperature for 10 mins; and then inactivated by the addition of Glycine to a final concentration of 125 mM. Fixed cells were then lysed in 1 ml ice-cold Farnham lysis buffer (0.5% NP-40, 85 mM KCl and 5 mM PIPES, pH 8.0), and this was then centrifuged at 16,000 *g* for 5 mins at 4^°^C. The supernatant was discarded and the nuclear pellet was resuspended in 1 ml ice-cold RIPA (Radioimmunoprecipitation Assay) buffer (1% NP40, 0.5% sodium deoxycholate, 0.1% SDS and PBS, pH 7.5). The nuclear lysates were sonicated on ice for 10 mins (20 × 30 sec bursts), and the shearing of the chromatin into 100–600 bp fragments was confirmed by agarose gel analysis. The preparation was then cleared of debris by centrifugation at 16,000 *g* for 5 mins at 4^°^C; and the supernatant transferred to a new tube. For each ChIP purification, the nuclear lysates were combined with 200 μl Dynabeads^®^ Sheep anti-Mouse (or Rabbit) IgG combined with 10 μg of either anti-FOXE1 [EPR6843] (Abcam, #ab134129) or anti-ELK1 [E277] (Abcam, #ab32106) precipitating primary antibody (or IgG negative control), and incubated overnight at 4^°^C. The following day, the beads were subjected to six 1 ml washes with ice-cold LiCl wash buffer (500 mM LiCl, 1% NP-40, 1% sodium deoxycholate, 100 mM Tris pH 7.5); and then a single wash with 1 ml ice-cold Tris-EDTA buffer (0.1 mM EDTA, 10 mM Tris-HCl, pH 7.5). The purified ChIP DNA was then eluted from the beads in 200 μl elution buffer (1% SDS, 0.1 M NaHCO_3_), incubated at 65^°^C for 1 hr. Finally, the DNA was reverse cross-linked by a further overnight incubation at 65°C and then purified using a PCR clean-up kit (Promega).

ChIP DNA was amplified using Qiagen’s HotStart Taq DNA polymerase, according to the manufacturer’s instructions. The primers sequences used to amplify regions of the *TERT* and *TPO* promoters can be made available upon request.

### Electro-mobility shift experiments

#### Expression and purification of recombinant proteins

Sub-confluent HEK293 cells grown in 6 cm petri dishes were transfected with a total of 10 μg of FOXE1 or ELK1 cDNA expression plasmid per plate. Forty-eight hrs later nuclear extracts were prepared using an NE-PER Extraction Kit (Thermo Scientific), according to the manufacturer’s instructions. The lysate was then combined with 50 μl Dynabeads^®^ Sheep anti-Mouse (or Rabbit) IgG combined with 1 mg of precipitating primary antibody; and the beads were washed as described previously (see co-immunoprecipitation method). The proteins of interest were eluted from the beads by the addition of 250 μl elution buffer (100 mM Glycine-HCl, pH 3.0) to the pelleted beads, which were then incubated at RT for 5 mins. Then, the sample was centrifuged at 16,000 g at 4^°^C for 5 mins, and the resulting supernatant transferred to a fresh pre-chilled 1.5 ml tube. The supernatant was then dialyzed overnight in pre-chilled 10 mM Tris-HCL (pH 7.5), using a Slide-A-Lyzer dialysis cassette (10 kDa MWCO; Thermo Fisher Scientific), and the sample then transferred to a pre-chilled 1.5 ml tube.

### DNA binding reaction and electrophoresis

For each binding reaction, 1 μg of purified protein was incubated at room temperature with biotin-labelled oligonucleotide probe comprising an 83 bp region of the human *TERT* gene promoter which contains the C228T and C250T mutations found in thyroid cancer (5′[Btn]CCCCGCCCCGTCCCGACCCCTCCCGGGTCCCCGGCCCAGCCCCCTCCGGGCCCTCCCAGCCCCTCCCCTTCCTTTCCGCGGCCCC-3′; the mutated nucleotide positions are underlined). To provide a positive control for FOXE1 DNA-binding a probe comprising the K region of the rat thyroglobulin promoter [42] was used; which contains a verified FOXE1 binding site (5′-[Btn]GAGGGAGTTCCTGTGACTA GCAGAGAAAACAAAGTGAGCCAC-3′). The protein and DNA were combined in binding buffer consisting of 150 mM KCl, 50 ng/μL Poly (dI-dC), 10% glycerol and 10 mM Tris (pH 7.5), and this was incubated at RT for 30 mins. The protein-DNA complexes were resolved by electrophoresis on a 6% polyacrylamide gel, electroblotted onto Biodyne B Nylon Membrane (Thermo Fisher Scientific), and then processed and detected using the LightShift^®^ Chemiluminescent EMSA Kit (Thermo Fisher Scientific).

### Statistical analyses

Differences in transcriptional activity/gene expression were analysed using Student’s *t-test* or one-way ANOVA.

## SUPPLEMENTARY MATERIALS FIGURES AND TABLE


