# Supplementary material for: Thyroid transcription factor FOXE1 interacts with ETS factor ELK1 to co-regulate TERT
Source: Oncotarget. 2016 Nov 11;7(52):85948–62. doi: 10.18632/oncotarget.13288 (PMC5349888; doi:10.18632/oncotarget.13288)
Supplement: Supplementary file 1 [file oncotarget-07-85948-s001.pdf]

Thyroid transcription factor FOXE1 interacts with ETS factor ELK1 to co-regulate TERT

Supplementary Materials

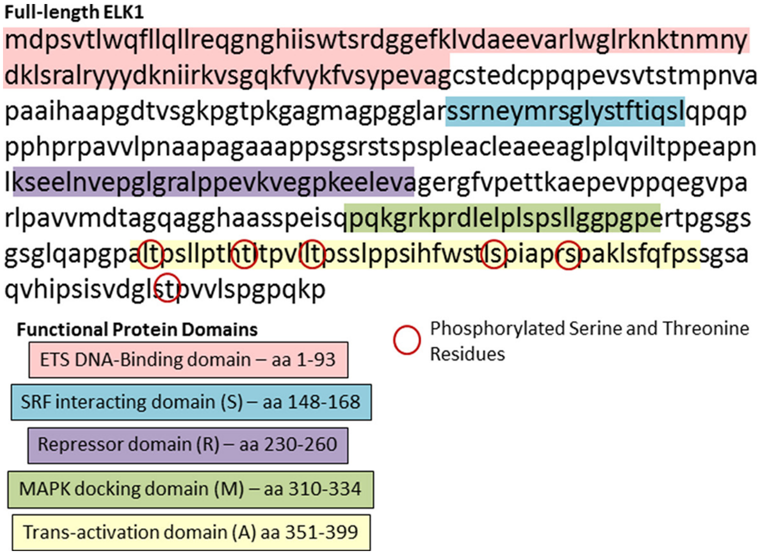

Supplementary Figure S1: Amino-acid sequence of the human ELK1 protein. Previously characterized functional domains and their amino-acid positions are highlighted. Serine and threonine residues known to be phosphorylated are also indicated.

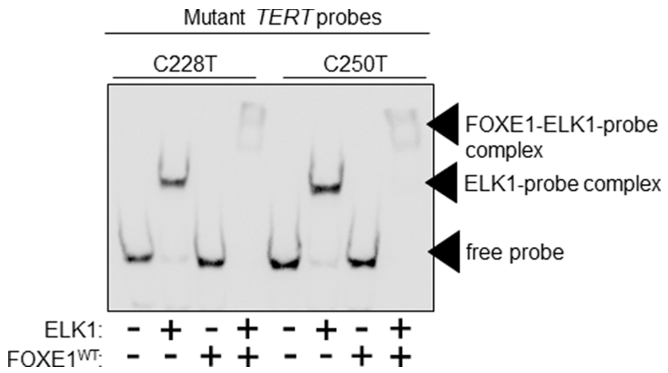

Supplementary Figure S2: FOXE1 and ELK1 interact with the mutant TERT gene promoters. Varying combinations of purified FOXE1-flag and ELK-HA proteins were incubated with either C228T or C250T *TERT* DNA-probe, resolved on a 6% polyacrylamide gels, and transblotted onto nylon membrane.

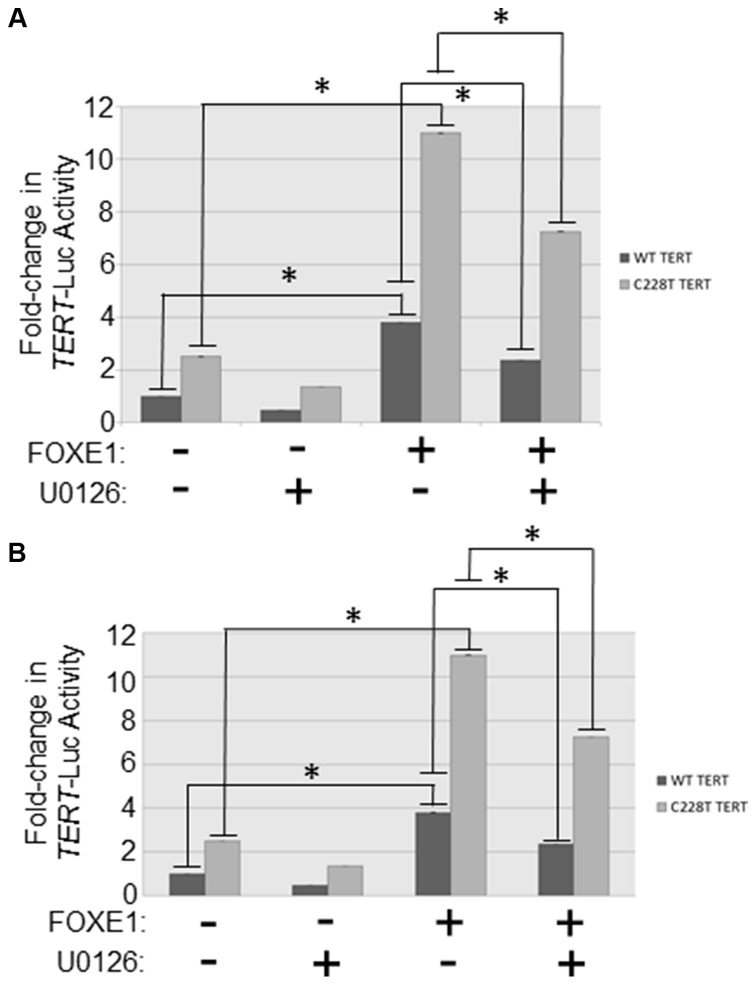

Supplementary Figure S3: FOXE1 regulates *TERT* transcription in ATC and non-tumorigenic thyroid cells. (**A**) SW1736 and (**B**) NThy cells were transiently transfected with either wild-type or C228T *TERT*-luc, and then different combinations of FOXE1-Flag or empty flag expression plasmids. Twenty-four hours post-transfection the cells were treated for a further 24 hours with 10 µM U0126 or vehicle, prior to whole cells lysates being harvested for luciferase reporter assays. Luciferase results are the mean (±SD) of three experiments, both performed in triplicate, expressed as fold increase in luciferase activity relative to empty vector transfected cells. Significant changes are highlighted (*$p < 0.05$, Student's *t*-test).

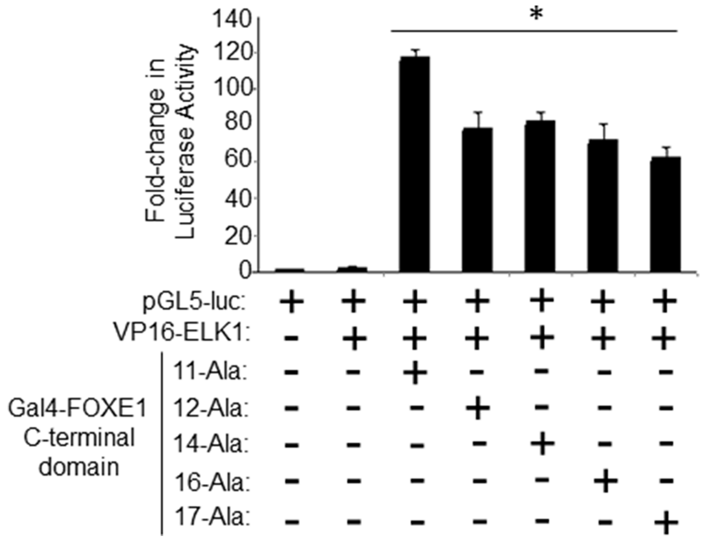

Supplementary Figure S4: The impact of polyalanine tract length upon FOXE1-ELK1 stimulated promoter activity. NThy cells were co-transfected with Gal4-VP16 responsive gene reporter, Gal4-FOXE1 plasmids with varying polyalanine tract size (0-17Ala) and VP16-ELK1. Cell lysates were harvested 48 hrs post-transfection and then reporter assays performed. Values are the the mean (±SD) of three experiments, each performed in triplicate, expressed as fold increase in luciferase activity relative to empty vector transfected cells (*$p < 0.01$, One-Way ANOVA).

Supplementary Table S1: Nucleotide sequences of primers used for PCR cloning and site directed mutagenesis. For the cloning primers, recognition sequences for EcoRI (GAATTC), BamHI (GGATCC), NotI (GCGGCCGC), XhoI (CTGAG), KpnI (GGTACC) and HindIII (AAGCTT) are underlined. For the mutagenic primers, mutated nucleotide residues are highlighted in bold.

**Cloning Primers**	
**Name**	**Nucleotide Sequence (5' to 3')**
Flag-FOXE1-F	TAATGAATTCTATGACTGCCGAGAGCGGGCCG
Flag-FOXE1-R	GACAGGATCCTCACATGGCGGACACGAA
HA-ELK1-F	TAATCTCGAGTTATGGACCCATCTGTGACG
HA-ELK1-R	GACAGCGGCCGCTCATGGCTTCTGGGGCCC
HA-ELK1-205-428-F	TAATCTCGAGTTGAGGCCTGTCTGGAGGCT
HA-ELK1-310-428-F	TAATCTCGAGTTCCGCAGAAGGGCCGGAAG
HA-ELK1-349-428-F	TAATCTCGAGTTGGGCCGGCGCTGACCCCA
HA-ELK1-1-309-R	GACAGCGGCCGCTCACTGGGAGATCTCAGGGCT
HA-ELK1-1-349-R	GACAGCGGCCGCTCACGGCCCCGGAGCCTGGAG
M2H-ELK1-F	TAATGGATCCGTATGGACCCATCTGTGACG
M2H-ELK1-R	GACAGGTACCTCATGGCTTCTGGGGCCC
M2H-FOXE1-CTERM-F	TAATGGATCCGTCCGGCTTACATGCACGACGCG
M2H-FOXE1-CTERM-R	GACAGGTACCTCAGTCGTGCATGTAAGCCGG
TERT-LUC-F	GGGGTACCCTGGCGTCCCTGCACCCTGG
TERT-LUC-R	CCCAAGCTTACGAACGTGGCCAGCGGCAG
**Mutagenic primers**	
Mut-TERT-C228T-F	CCCCGCCCCGTCCCGACCCCTCCCGGGTCCCCGGCCCAGCCCC**T**TCCGGG
Mut-TERT-C228T-R	CCCGGA**A**GGGGCTGGGCCGGGGACCCGGGAGGGGTCGGGACGGGGCGGGG
Mut-TERT-C250T-F	CCCCGCCCCGTCCCGACCCCT**T**CCGGGTCCCCGGCCCAGCCCCCTCCGGG
Mut-TERT-C250T-R	CCCGGAGGGGGCTGGGCCGGGGACCCGG**A**AGGGGTCGGGACGGGGCGGGG
Mut-ELK1-S>A-383-F	CTCCTAGCATTCACTTCTGGAGCACCCTG**G**CTCCCATTGCGCCCCGT
Mut-ELK1-S>A-383-R	ACGGGGCGCAATGGGAG**C**CAGGGTGCTCCAGAAGTGAATGCTAGGAG
Mut-ELK1-T>A-353-368-F	CCGGCGCTG**G**CCCCATCCCTGCTTCCTACGCATACATTG**G**CCCCGGTGCTGCTG**G**CACCC
Mut-ELK1-T>A-353-368-R	GGGTG**C**CAGCAGCACCGGGG**C**CAATGTATGCGTAGGAAGCAGGGATGGGG**C**CAGCGCCGG
Mut-ELK1-T>A-417-F	GGCCTCTCG**G**CCCCCGTGGTGCTCTCCCCAGGGCCCCAGAAGCCATGAGCGGCCGCTGTC
Mut-ELK1-T>A-417-R	GACAGCGGCCGCTCATGGCTTCTGGGGCCCTGGGGAGAGCACCACGGGGG**C**CGAGAGGCC
